# Application of Tendon-Derived Matrix and Carbodiimide Crosslinking Matures the Engineered Tendon-Like Proteome on Meltblown Scaffolds

**DOI:** 10.1155/term/2184723

**Published:** 2025-02-26

**Authors:** Thomas Lee Jenkins, Sadhana Venkataraman, Aya Saleh, Sarah Calve, Behnam Pourdeyhimi, Dianne Little

**Affiliations:** ^1^Weldon School of Biomedical Engineering, Purdue University, West Lafayette, Indiana, USA; ^2^Department of Mechanical Engineering, University of Colorado-Boulder, Boulder, Colorado, USA; ^3^The Nonwovens Institute, North Carolina State University, Raleigh, North Carolina, USA; ^4^Department of Basic Medical Sciences, College of Veterinary Medicine, Purdue University, West Lafayette, Indiana, USA

**Keywords:** biomaterials, collagen, extracellular matrix, human adipose stem cells, mesenchymal stem cell

## Abstract

**Background:** Tendon injuries are increasingly common and heal by fibrosis rather than scar-less regeneration. Tissue engineering seeks to improve repair using synthetic polymer scaffolds with biomimetic factors to enhance the regenerative potential.

**Methods:** In this study, we compared three groups, namely, poly(lactic acid) (PLA) meltblown scaffolds, PLA meltblown scaffolds coated with tendon-derived matrix (TDM), and PLA meltblown scaffolds with carbodiimide crosslinked TDM (2.5:1:1 EDC:NHS:COOH ratio) (EDC-TDM) and determined their potential for engineered tendon development. We cultured human adipose stem cells (hASCs) for 28 days on meltblown scaffolds (*n* = 4–6/group) and measured tensile mechanical function, matrix synthesis, and matrix composition using biochemical assays and proteomics.

**Results:** Coating PLA meltblown scaffolds with TDM improved yield stretch and stress at 28 days compared with PLA. Matrix synthesis rates for TDM or EDC-TDM were similar to PLA. Proteomic analysis revealed that hASCs produced a collagen-rich extracellular matrix, with many tendon-related matrix proteins. Coating scaffolds with TDM led to an increase in collagen type I whereas EDC-TDM scaffolds had an increase in glycoproteins and ECM regulators compared with other groups, consistent with increased maturity of the newly deposited matrix.

**Conclusions:** TDM coating and crosslinking of meltblown scaffolds demonstrated matricellular benefits for the proteome of engineered tendon development but provided fewer clear benefits toward mechanical, biochemical, and rate of matrix accumulation than expected, and that previous work with electrospun scaffolds would suggest. However, electrospun scaffolds have different fiber structure and microarchitecture than meltblown, suggesting that further consideration of these differences and refinement of TDM application methods to meltblown scaffolds is required.

## 1. Background

There are more than 545,000 rotator cuff repair surgeries performed each year in the US which heal by fibrosis and have retear rates up to 94% for larger tears [1–3]. The fibrotic tissue that forms after repair is characterized by a disorganized collagen matrix—unlike healthy tendon that relies on a highly aligned hierarchical structure of mostly collagen fibers to transmit force from muscle to bone [4, 5]. Augmenting the repair with a patch is a strategy aimed at improving repair rates, but current patches have mixed efficacy, with slow cell infiltration, low remodeling, and mechanical properties that do not provide adequate support in the initial postoperative period [6, 7]. Tissue engineered scaffolds seek to address these gaps and improve tendon regeneration and repair. Tissue engineering seeks to employ biomaterials, stem cells, and biomimetic factors [8] to improve repair and stimulate regeneration. In tendon tissue engineering, polymer fiber scaffolds provide microarchitectural cues similar to collagen fibers. Electrospinning is a commonly used technique to produce such scaffolds and allows stem cells to grow and differentiate toward tendon-like cells producing tendon-like extracellular matrix enriched in aligned collagen type I [9–16].

Electrospinning is a process where polymer in solution is extruded through a high-voltage electric field toward a grounded collector. Electrospinning is commonly investigated in tendon tissue engineering because it allows a high degree of control over the structure and the architecture (fiber diameter, alignment, and porosity) [17, 18]. However, single-needle electrospinning can be quite low throughput, calculated to accumulate at a rate of 0.8–82.3 mm^3^/min [10, 17, 19, 20]. Developments have been made to improve the throughput of electrospinning. For example, multineedle electrospinning can produce scaffolds at a much higher rate but can struggle with needle blockage. Needleless electrospinning setups are being developed that improve throughput [21, 22]. However, the single-needle electrospinning set up used in our lab takes several hours to produce a clinically sized scaffold and requires toxic solvents to dissolve the polymer [10, 19].

In contrast, meltblowing is a process where a melted polymer is extruded through a series of orifices (die) where two converging streams of high velocity, hot air jets attenuate the fibers at velocities approaching 280 m/s. The fibers undergo a whipping motion during their flight to the collector and form a self-bonded structure similar to electrospinning. The fibers self-quench and solidify due to their high surface area and if needed, there is a secondary quench that can cool down the fibers to help solidify and crystallize the structure. Meltblowing is a high-throughput process—with fabrics produced at rates of up to 5000 m/min (100 kg/hr/m). Meltblowing is a high-throughput process—with fabrics produced at rates of up to 5000 m/min (100 kg/hr/m) [23]. Meltblowing is commonly used for producing N95 facemasks, barriers in medical gowns, and filtration and food packing materials [24], with some recent investigations in tissue engineering [25–34]. In our earlier studies, we found similar tendon-like matrix production and aligned collagen compared to previous electrospun studies [31], despite the fact that meltblown fabrics have a larger fiber diameter (9.4 μm (7.8–11.5) vs. 1.76 μm (1.06–2.58)) and the fibers are ∼60% less aligned than electrospun scaffolds as measured by fast Fourier transform of fibers imaged in scanning electron micrographs [19, 31]. The meltblowing apparatuses are easy to modulate and adapt the die heads, surfaces, and airflow to alter fiber diameter, alignment, and porosity—at industrial scale which can ease the potential for future translation into clinical devices. Recent advances to the meltblowing technology can achieve randomly oriented to highly aligned fibers in the 1–100 μm range with the ability to mimic the shape of human body parts at ∼20X the speed of electrospinning [33]. Future work continuing after the current study will aim to optimize meltblowing for tendon tissue engineering.

We and others have shown that tendon-derived matrix (TDM) stimulates engineered tendon-like development by various cell types, both in vitro [10, 35–38] and in vivo [35, 39, 40]. However, unseeded TDM-coated electrospun scaffolds lost TDM during culture in vitro, which is a concern for translation [10]. More recently, carbodiimide crosslinking to retain TDM to electrospun scaffolds also increased de novo collagen synthesis and improved stiffness and mechanical function [36]. Therefore, the objective of this study was to evaluate how TDM coating or carbodiimide crosslinking TDM to poly(lactic acid) (PLA) meltblown scaffolds modulated tendon-like matrix production by human adipose-derived stem cells (hASCs), with the hypothesis that TDM would enhance new extracellular matrix synthesis, particularly if the TDM was retained on the scaffold through carbodiimide crosslinking.

## 2. Materials and Methods

### 2.1. TDM

Flexor and extensor tendon was harvested from adult female porcine hind limbs obtained from a slaughterhouse. The tissue was minced, lyophilized, and pulverized (6750 Spex SamplePrep Freezer Mill; Spex CertiPrep) before being placed in a progressive series of sieves to pass through a 75-μm wire mesh. The sieved powder was stored at room temperature until use.

### 2.2. Meltblown Scaffold Preparation

PLA meltblown nonwoven fabrics were produced at the NonWovens Institute (North Carolina State University) at 40 kg/hr/m and 1400 m^3^/h/m of air by using 6100D PLA (Natureworks, Minneapolis, MN) [31]. The PLA meltblown scaffolds have a median fiber diameter of 9.4 μm (7.8 μm and 11.5 μm) with a thickness of 0.25 ± 0.03 mm [31]. Porosity has not been measured, but hASCs infiltrate the full thickness of the scaffold and produce a robust extracellular matrix within 28 days of culture [31]. Scaffolds to be used for biochemical assays and histology were cut into 0.5 × 3 cm rectangles. The long axis of the scaffold paralleled the long axis of the meltblown fabrics so that the fibers aligned along the long axis of the scaffold [31]. Scaffolds to be used for mechanical testing were cut into a dog-bone shape and stained with India ink in the center and at 5 mm proximal and distal to allow for regional strain analysis. All scaffolds were rehydrated and sterilized in a graded series of ethanol washes before a final wash in PBS (Gibco 10,010–031). Scaffolds were separated into three groups: no treatment, TDM (TDM) coated, or carbodiimide crosslinked TDM coated (EDC-TDM). Scaffolds were coated by direct pipetting with TDM in PBS to obtain 2 mg TDM/cm^2^. The carbodiimide group was crosslinked at a ratio of 2.5:1:1 1-ethyl-3-(3-dimethylaminopropyl) carbodiimide hydrochloride (EDC) to N-hydroxysuccinimide (NHS) to COOH (collagen) [36].

### 2.3. Cell Seeding and Culture

Both sides of each scaffold were sterilized under UV radiation for 10 min and prewetted with 25 μL of media. hASCs previously isolated by collagenase digestion of the stromal vascular fraction of lipoaspirate surgical waste from three deidentified female donors (age: 36–59 and body mass index: 19.6–33.1) with the approval of the Duke University Institutional Review Board were used at Passage 3 [41]. hASCs were not sorted for canonical cell surface markers [42] but have demonstrated multipotency for adipogenesis, osteogenesis, chondrogenesis, and tenogenesis [10, 43]. Cells were seeded at a density of 5 × 10^5^ hASCs/cm^2^ on each side by pipetting and were allowed 15 min for attachment, after which the scaffolds were reversed, and the process was repeated. Scaffolds were maintained in 6-well plates coated with 2% agarose without growth factors at 37°C and 5% CO_2_ in Advanced DMEM (Life Technologies) supplemented with 10% fetal bovine serum, 1% penicillin-streptomycin-fungizone (Life Technologies), 4 mM L-glutamine (Life Technologies), and 15 mM L-ascorbic acid-2-phosphate (Sigma-Aldrich). Media was changed three times a week for the first 2 weeks and every other day for the final 2 weeks of culture.

### 2.4. Histology

On days 0 (4 h postseeding) and 28, unseeded and hASC-seeded scaffolds (*n* = 5) were harvested and embedded in optimal cutting temperature gel (Sakura) and frozen at −80°C. 10 μm sections were mounted on slides. Slides were stained with either picrosirius red or hematoxylin and eosin and Safranin-O/Fast Green and evaluated using an Olympus BX53 Microscope (Olympus Life Science). Slides stained with picrosirius red were also evaluated with polarized light microscopy to investigate the alignment of fibrillar collagen [31]. For all polarized light microscopy, the scaffolds' fibers are aligned horizontal to the microscope stage, which corresponds to a 45° angle of the polarizer.

### 2.5. Mechanical Testing

Mechanical testing samples (*n* = 6), unseeded and hASC seeded, were harvested on days 0 and 28 and wrapped in gauze soaked in PBS and stored at −80°C. Initial scaffold thickness was measured using an Emergent HR2000-M camera (Emergent Vision Technologies) and digital calipers in ImageJ. Scaffolds were placed in custom-built clamps with 80-grit sandpaper glued to the inside edge of the clamp. The specimens were tested at a 1%/s strain rate with a 0.5 g preload using an electromechanical testing system (Acumen 3, MTS) with a 10 N load cell (MTS 661.09C-30). Midsubstance strains were calculated from digital images acquired at 50 Hz and interpolated to load frame data using custom MATLAB code (MATLAB R2019b). The transition stretch was calculated using a bilinear curve fit to determine the toe-region [44]. The linear-region modulus, stiffness, and the stretch and stress at yield were calculated in Microsoft Excel (Microsoft Office).

### 2.6. Noncanonical Amino Acid Labeling

New matrix synthesis on scaffolds from each group (PLA, TDM, and EDC-TDM) was evaluated using noncanonical amino acid labeling. L-Azidohomoalanine (AHA) (Click Chemistry Tools, 1066) was used to label new proteins [45, 46] at various stages in the culture period. A control treatment supplying methionine (Sigma M9625) instead of AHA was used for comparison. Scaffolds were cut into 3 cm × 0.5 cm rectangles (*n* = 5/treatment/group/time point) and prepared as described in Section 2.2. Scaffolds were seeded using hASCs at a density of 5 × 10^5^ hASCs/cm^2^ and cultured in 6-well plates (Falcon 351,147) suspended between Teflon rings with 4 mL of media per well. Two types of media were used: a media supplied with methionine and a media with AHA instead of methionine. Both media types had a base composed of methionine free DMEM (Gibco 21,013,024), 10% Hyclone FBS, 1% antibiotic-antimycotic (Gibco 15,240,062), 1% GlutaGRO (Corning 25015Cl), 63 mg/mL L-Cystine (Sigma 30,200), 15 mM L-ascorbic acid-2-phosphate (Sigma-Aldrich). Both the media had a final concentration of either 40 μg/mL L-methionine or 40 μg/mL AHA in the base media for the methionine-control and AHA-treatment media, respectively. Scaffolds were cultured for 28 days before harvest. AHA media were only supplemented instead of methionine media to treated samples during the last 7 day of cell culture. This 7-day culture period was selected because hASCs did not survive a 2-week or longer treatment period with AHA media but did survive up to 7 days, as seen by other groups for bone marrow-derived mesenchymal stem cells (MSCs) and chondrocytes [47–49]. Scaffolds were fed 1 mL of the specified media every 2 days for the time points in the first 2 weeks or every day in the third and fourth weeks of culture. At harvest, the scaffolds were incubated with 30 μM Alexa Fluor 647 dibenzocyclooctyne-amine (AFDye 647 DBCO, Click Chemistry Tools, 1302) for 30 min at 37°C and 5% CO_2_. Scaffolds were transferred into glass bottom dishes (MatTek P35G-1.5-10-C), counterstained with DAPI (300 nM, Invitrogen D1306) for 5 min, washed with PBS, and stored in the dark at 4°C prior to imaging.

Scaffolds were imaged on a Nikon A1R-MP confocal microscope using z-stacks throughout the full thickness of the scaffold (∼150–200 μm) at intervals of 6.25 μm per slice at 10X magnification and over 25 μm thickness at intervals of 1 μm per slice at 40X. Projections in each of the three planes were made in FIJI (NIH, Version 2.1.0). Images were analyzed using a custom MATLAB code (MATLAB R2019b) to calculate the volume of protein and nuclei and normalize the volume of protein per volume of nuclei for comparison of protein production.

### 2.7. Biochemical Assays

On days 0 and 28 of culture, unseeded and hASC-seeded scaffolds were harvested and lyophilized to obtain dry weight. The scaffolds were minced and digested in papain (125 μg/mL) at 60°C for one week. The Picogreen Assay was used to quantify dsDNA content [31, 41]. The sulfated glycosaminoglycan (sGAG) content was quantified using 1,9-dimethylmethylene blue dye (pH 3.0) and analyzed spectrophotometrically [50]. The total collagen content was determined by the hydroxyproline assay using a conversion factor of 1:7.46 to convert hydroxyproline to collagen [31, 51]. Total protein was quantified using a Pierce BCA Protein Assay Kit (ThermoFisher, #23227) after an overnight 4:1 acetone incubation at −20°C to precipitate proteins, then 10 min centrifugation at 21,000 RPM, acetone was removed, and the pellets dried for 30 min in a fume hood and resuspended in dH_2_O before using the BCA Assay kit as manufacturer directions. All results were normalized to scaffold dry weight.

### 2.8. Proteomics

The samples remaining after sectioning for histology were washed twice in 50 mM ammonium bicarbonate for 10 min each before being stored at −80°C freezer overnight. Samples were then cut into 2 × 2 mm squares and rinsed in 50 mM ammonium bicarbonate for 10 min then frozen and lyophilized (Labconco FreeZone 4.5L −105°C Benchtop Freeze Dryer). The samples were pulverized (6775 Freezer Mill; Spex SamplePrep) with a 5-min precool, 3 cycles of 1.5 min runs at 10 Hz, and 2-min cooling periods in between runs. The pulverized samples were rinsed from the microvial (Spex 6757) using 50 mM ammonium bicarbonate and lyophilized.

Proteins were extracted by using 0.1% RapiGest (Waters) in 50 mM ammonium bicarbonate and sonicated on ice 3 times for 10 s at 40% power, with 30 s in between each sonication. The samples were then heated for 10 min at 80°C, centrifuged at 21,000 RPM for 10 min, and the supernatant was collected. A total of 250 μL of 0.1% RapiGest was added to the pellet, the process was repeated, and the supernatants were combined and vortexed. Extracted proteins were quantified via Pierce BCA Protein Assay Kit (ThermoFisher #23225). 40 μg of protein was placed into a new centrifuge tube, and 4X volume of cold acetonitrile was added to each tube and left at −20°C overnight. Supernatant was removed and evaporated in the Vacufuge Plus (Eppendorf). 20 μL of LDS NuPAGE buffer (ThermoFisher NP002) was added to each sample and mixed in a thermomixer for 10 min at 70°C and 750 rpm. In order to avoid overwhelming the columns with PLA contamination, samples were run in NuPAGE Bis-Tris gel (ThermoFisher NP0301) for < 2 min in a Mini Gel Tank (Invitrogen A25977) until the gel had left the well. Then, the entire gel band for each protein sample was cut into 1 × 1 mm squares and de-alkylated in 10 mM dithiothreitol in 25 mM ammonium bicarbonate for 1 h in the thermomixer at 55°C and 550 rpm, followed by a 45-min incubation at room temperature in the dark in 55 mM iodoacetamide in 25 mM ammonium bicarbonate. Samples were then taken through a 25 mM ammonium bicarbonate to acetonitrile wash, before being dried out in the speed vac. Next samples were incubated at 37°C overnight in the thermomixer at 450 rpm with 1.6 mg of Trypsin/Lys-C mix (Promega V5071) in 25 mM ABC to digest the proteins. Digested peptides were extracted by sonicating sample in 5% TFA in 60% ACN for 15 min and removing the solution twice, before drying out in the speed vac. Samples were resuspended in 3% ACN and 0.1% formic acid, with 10 fmol/μL Yeast alcohol dehydrogenase (ADH) (Waters 186,002,328) as a standard and loaded into the Lumos Fusion Orbitrap for analysis. One μg of peptide was loaded into the column for each sample and run for 190 min.

The data files were processed using the MaxQuant computational proteomics platform version 1.6.7. Spectra were searched against SwissProt (https://www.uniprot.org) reference proteomes *Homo sapiens* (ID: UP000005640) and *Sus scrofa* (ID: UP000008227), as well as surrogate standard ADH1_YEAST (ID: P00330). Intensity values for the top 3 peptides for each protein identified within each sample were averaged. Top 3 averages were normalized to ADH1_YEAST to estimate mol/ng of protein in each sample [52]. The mass of each protein was normalized to total protein within each sample. Proteins identified were aligned with the Matrisome Project Database [53] and comparisons were made between different extracellular matrix protein groups and for nonmatrisomal proteins.

### 2.9. Statistics

Data are reported as the mean ± SD or median (25th quartile and 75th quartile) and evaluated for the effect of seeding, time, and treatment. We tested data for normality using the Shapiro–Wilk test and equal variance using the Brown–Forsythe test. Parametric data were evaluated using factorial analysis of variance (ANOVA). The Tukey post hoc test was used to determine differences between treatments following ANOVA. We compared nonparametric data using the Kruskal–Wallis or the Friedman test followed by Wilcoxon each pair post hoc testing. Significance was reported at the 95% confidence level for all analyses (*α* = 0.05). All statistics were calculated using JMP 16.1.0 (SAS Institute).

## 3. Results

### 3.1. Histology

**Figure 1:**
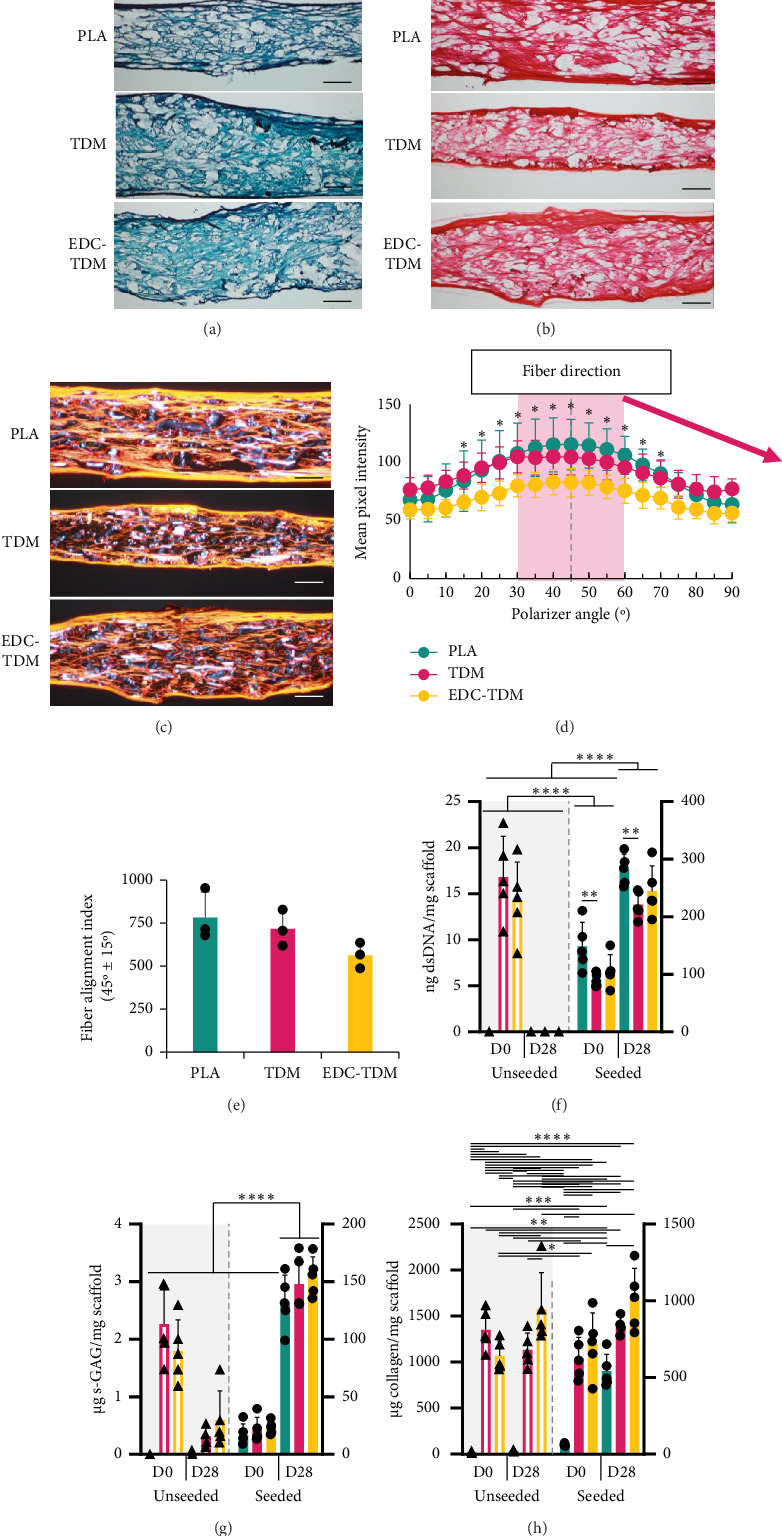
hASC histology and matrix content on meltblown scaffolds. Safranin-O/fast green staining under visible light (a), picrosirius red staining under visible light (b), and orange/red birefringence observed under polarized light (c) for untreated (PLA), tendon-derived matrix-coated (TDM), and TDM-crosslinked (EDC-TDM) poly(lactic acid) meltblown scaffolds cultured for 28 days with hASCs. Scale bar represents 100 μm. Mean ± SD pixel intensity of orange/red birefringence at each assessed polarizer angle, with 45° (d) (*n* = 3) corresponding to the direction of the collector of the meltblowing apparatus. Mean ± SD fiber alignment index (e) (sum of pixel intensity from 45° ± 15° for each treatment group. ⁣^∗^Difference between mean pixel intensity and intensity at 0° polarizer angle for all blends, (*p* < 0.05, repeated measures ANOVA, Tukey post hoc test). Mean ± SD dsDNA (f), sulfated glycosaminoglycan (s-GAG) (g), and collagen (h) content of poly(lactic acid) meltblown scaffolds that were untreated (PLA), were coated with tendon-derived matrix (TDM), or had tendon-derived matrix crosslinked to the scaffold (EDC-TDM) 0 and 28 days after seeding with 5 × 10^5^ hASC/cm^2^ (*n* = 5; ^∗^*p* < 0.05, ^∗^^∗^*p* < 0.01, ^∗∗∗^*p* < 0.001, ^∗∗∗∗^*p* < 0.0001; ANOVA; Tukey's post hoc test).

Staining of hASC-seeded scaffolds after 28 days with safranin-O/fast green (Figure 1(a)) demonstrated collagen throughout the thickness of the scaffold, with undetectable amounts of proteoglycans, as expected for tendon ECM. The picrosirius red stain (Figure 1(b)) under polarized light (Figure 1(c)) showed much of the collagen produced to be aligned through the full thickness of the scaffold. Fast Fourier transform analysis revealed the collagen aligned with the direction of the underlying fibers (Figure 1(d)), and the fiber alignment index (Figure 1(e)) showed that there was no significant difference in collagen alignment between the groups. The Day 0 samples, as well as the Day 28 unseeded scaffolds, detach from the slides during the initial PBS wash to remove the OCT and were not included in analyses.

### 3.2. Biochemical Assays

When cultured in media optimized for engineered tendon development (Section 2.3), dsDNA (Figure 1(f)) increased significantly over 28 days in culture in seeded scaffolds, with negligible amounts in unseeded scaffolds at Day 28. PLA-seeded scaffolds had more dsDNA than TDM but not EDC-TDM scaffolds at both Day 0 and Day 28, while there was no difference between TDM and EDC-TDM at either time point. The sGAG content increased over 28 days in culture for seeded scaffolds (Figure 1(g)), with no difference between treatment groups for sGAG content at either time point. In unseeded scaffolds, TDM and EDC-TDM scaffolds had greater collagen than unseeded PLA scaffolds at both time points (Figure 1(h)). In seeded scaffolds at day 0, the collagen content for TDM scaffolds was not different than PLA alone, or compared with EDC-TDM, but the collagen content of EDC-TDM was greater than PLA alone. By Day 28, the collagen content was greater on TDM and EDC-TDM scaffolds than on PLA scaffolds, but only EDC-TDM-seeded scaffolds significantly increased collagen content over 28 days in culture.

### 3.3. Mechanical Properties

**Figure 2:**
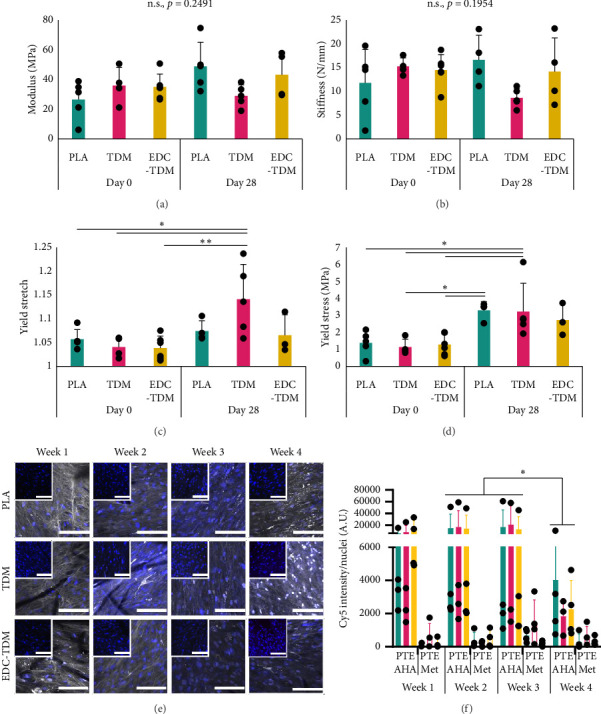
hASC-seeded meltblown scaffold mechanical properties and new matrix synthesis. Mean ± SD linear-region modulus (a), stiffness (b), yield stretch (c), and yield stress (d) in hASC-seeded untreated (PLA), TDM-coated (TDM), and TDM-coated, carbodiimide crosslinked (EDC-TDM) PLA meltblown scaffolds after 0 and 28 days of culture (*n* = 4–6). hASC: human adipose-derived stem cell. TDM: tendon-derived matrix. Representative confocal maximum Z projection images (e) for untreated (PLA), tendon-derived matrix-coated (TDM), and TDM-crosslinked (EDC-TDM) poly(lactic acid) meltblown scaffolds cultured for 28 days with azidohomoalanine (AHA) in the media; white signal represents AHA; blue represents nuclei (DAPI). Inserts in upper left show methionine (Met) control samples. Scale bar = 100 μm. Mean ± SD of Cy5 signal (measuring AHA) per nucleus (f) in each sample. P = PLA; T = TDM; E = EDC-TDM (*n* = 5) (^∗^*p* < 0.05, ^∗^^∗^*p* < 0.01, ^∗∗∗^*p* < 0.001, ^∗∗∗∗^*p* < 0.0001; ANOVA; Tukey's post hoc test).

Overall, at Day 0, the unseeded scaffolds had a greater linear-region modulus, yield stress, and stiffness than the seeded scaffolds with no difference in yield stress (Supporting Table 1). Many of the unseeded scaffolds degraded and disintegrated during harvest at Day 28 before testing, as seen previously [31]. Thus, there was insufficient sample size to evaluate for the effect of treatment group EDC-TDM (*n* = 0), PLA (*n* = 3), and TDM (*n* = 2) or interactions with other factors. When combined, at Day 28, the residual unseeded scaffolds had greater linear-region modulus and stiffness than seeded scaffolds, but seeded scaffolds had greater yield stretch and yield stress (Supporting Table 1). In contrast, in seeded scaffolds, the modulus (Figure 2(a)) and stiffness (Figure 2(b)) of the linear region were maintained over 28 days in culture. With regard to seeded scaffold type, for EDC-TDM scaffolds, yield stretch and yield stress were maintained over 28 days in culture. In contrast, yield stretch (Figure 2(c)) increased over 28 days in culture for the hASC-seeded TDM scaffolds and yield stress (Figure 2(d)) increased over 28 days in culture for the hASC-seeded PLA and TDM scaffolds. The tangent modulus at 4% strain (Supporting Table 2) was not different with seeding at Day 0 (*p*=0.4203), over time in seeded scaffolds (*p*=0.9986), or between treatment groups in seeded (*p*=0.9953) or unseeded (*p*=0.8766) scaffolds. Seeded scaffolds exhibited a toe region in stress–stretch curves (Supporting Figure 1) at less than 1% stretch. The transition stretch (Supporting Table 3) was not different with seeding at Day 0 (*p*=0.4110), over time in seeded scaffolds (*p*=0.6601), or between treatment groups in seeded (*p*=0.2994) or unseeded (*p*=0.5122) scaffolds.

### 3.4. Noncanonical Amino Acid Labeling

All treatment groups exhibited new matrix synthesis during weeks 1, 2, 3, and 4 of culture (Figure 2(e)). As expected, during all 4 weeks, the AHA-fed scaffolds had significantly higher Cy5 signal than the methionine fed scaffolds (*p* < 0.0001). In the AHA-fed scaffolds, there was no difference between treatments (PLA vs. TDM vs. EDC-TDM) in the rate of matrix synthesis per cell as assessed by Cy5 intensity (Figure 2(f)) during weeks 1–3 (Week 1 *p*=0.3395; Week 2 *p*=0.5723; and Week 3 *p*=0.1936), although there was a trend toward a difference (PLA > TDM, *p*=0.0822) due to scaffold group in Week 4. New matrix incorporation was significantly less during Week 4 compared and Weeks 2 and 3 (*p*=0.0361). hASCs were observed throughout the full thickness of the scaffold in all weeks of culture.

Scaffolds cultured for noncanonical amino acid labeling used DMEM supplemented with cysteine and AHA or methionine (2.6), rather than advanced-DMEM supplemented with L-glutamine and L-ascorbate levels optimized for maximal collagen production as for other experiments, so we repeated biochemical assays (Supporting Figure 2A–2C) for these samples and compared them to prior results reported in Section 3.2 (Figures 1(f), 1(g), and 1(h)). Total protein, measured by BCA assay, showed an increase in protein over 4 weeks in culture in all treatment groups, whether given AHA or methionine in the media (Supporting Figure 2A and 2D). There was no difference in total protein between any treatment group within any week of culture. Total dsDNA increased similarly over time (Supporting Figure 2B and 2E), with no difference between treatments at any time point. After 2 weeks of culture there were no further increases in DNA content for PLA, but the DNA content continued to increase for TDM and EDC-TDM through the full culture period. Collagen content—approximated by the hydroxyproline assay—was higher in the EDC-TDM treatment group at Week 1 (Supporting Figures 2C and 2F) and higher in the TDM group at Week 3 compared with PLA for AHA treated scaffolds but, otherwise, there was no significant effect of time or scaffold. sGAGs increased from Week 1 to Week 2 but did not increase over time during Week 3 or 4 (data not shown). Within each week, there was no difference between the PLA, TDM, or EDC-TDM scaffolds in the amount of sGAG (*p* > 0.05). There was no difference in DNA, protein, or collagen content between the methionine controls and the AHA-fed scaffolds for any treatment group or timepoint (*p* > 0.05). Overall, scaffolds cultured in noncanonical amino acid labeling media, with AHA or methionine supplementation, lost the effect of treatment on dsDNA and collagen seen in scaffolds cultured in advanced DMEM (Figures 1(f), 1(g), and 1(h)).

### 3.5. Proteomics

A total of 8928 peptides (Supporting Table 4) and 956 proteins (Supporting Table 5) were identified in the day 28 samples. The 772 proteins remaining after contaminants and cDNA were removed (Supporting Table 6) were additionally categorized using the Matrisome Project [53]. The 772 proteins identified categorized into 91 core matrisome proteins, 45 matrisome-associated proteins, and 636 nonmatrisomal proteins (Supporting Table 6).

#### 3.5.1. Fold Change Analysis

Fold change analysis of the intensity measurements of seeded, Day 28 scaffolds between EDC-TDM and PLA revealed 8 differentially expressed core matrisome proteins and 16 matrisome-associated proteins (Table 1; Supporting Table 7). For TDM compared with PLA, collagen type V was the only matrisome protein upregulated in TDM. In EDC-TDM compared with TDM-seeded scaffolds, 1 core-matrisome protein and 6 matrisome-associated proteins were upregulated (Table 1). For nonmatrisomal proteins, there were 181 differentially expressed proteins between PLA-, TDM-, and EDC-TDM-seeded scaffolds (Supporting Table 7), many related to cytoskeletal organization and cell motility (Supporting Table 14). For TDM scaffolds, the seeded scaffolds had a total of 180 significantly different proteins compared with unseeded (Supporting Table 9), which include enriched pathways (Supporting Table 16) for collagen biosynthesis (GO:0032964) and collagen fibril organization (GO:0030199). For EDC-TDM scaffolds, there were 444 significantly different proteins in the seeded scaffolds compared with unseeded (Supporting Table 9), which included enrichment for collagen fibril organization (GO:0030199) and collagen metabolic process (GO:0032963) (Supporting Table 17).

#### 3.5.2. Top3 Quantification—Overview

**Figure 3:**
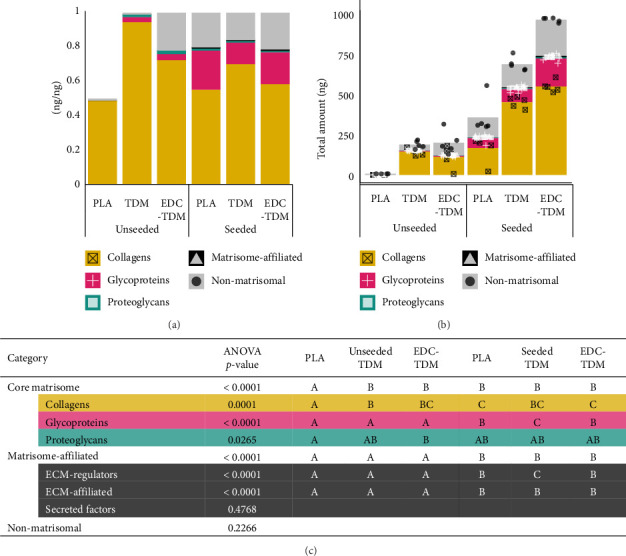
Matrix composition in hASC-seeded meltblown scaffolds. Mean percentage (a) and total amount (b) of each matrisome category for unseeded and hASC-seeded, untreated (PLA), tendon-derived matrix-coated (TDM), and TDM-crosslinked (EDC-TDM) poly(lactic acid) meltblown scaffolds after 28 days in culture. Groups with different letters in the table (c) are significantly different from each other by percentage (*p* < 0.05, ANOVA; Tukey's post hoc test).

Compared to unseeded scaffolds, all scaffolds cultured with cells for 28 days exhibited a proteome that was mostly collagen, ranging from ∼45% to 62% collagen by dry weight (Figures 3(a) and 3(b); Supporting Table 8). The total amount of protein identified was several orders of magnitude lower in the PLA unseeded samples (0.25 ± 0.49 ng) than in the TDM or EDC-TDM unseeded (∼175 ng) or any seeded scaffolds (350–950 ng) (Figure 3(b)). However, 2 out of 4 unseeded PLA scaffolds contained protein, likely relating to contamination during previous cryosectioning for histological examination or during fabrication in an industrial pilot manufacturing system, rather than a clean room as would occur in future translational application. Thus, the absence of protein in half the PLA samples results in a reduction in the ng/ng quantification of protein to 0.5 rather than 1.0 in unseeded PLA samples (Figure 3(a)). Incorporating TDM increased the total protein synthesized, as assessed using this Top3 method of quantification. The seeded TDM (∼650 ng) and EDC-TDM (∼950 ng) scaffolds had greater amounts of protein than the sum of the unseeded TDM/EDC-TDM (∼175 ng) and the seeded PLA (∼350 ng). In the seeded scaffolds, 104 proteins were significantly different quantitatively between the PLA, TDM, and EDC-TDM scaffolds including 18 matrisome proteins (Table 2) and 86 nonmatrisomal proteins (Supporting Table 8). GO Analysis revealed enriched pathways of cytoskeleton organization and cell adhesion (Supporting Table 15). TDM- and EDC-TDM-seeded scaffolds had 331 and 472 proteins with increased expression compared with unseeded, respectively (Supporting Table 10), with tendon development (GO:0035989) identified as a highly enriched pathway, along with collagen metabolic process (GO:0032963) and collagen fibril organization (GO:0030199) (Supporting Table 18 and Supporting Table 19). At Day 28, in seeded scaffolds, there was no difference between treatment groups in the amount of collagen (*p*=0.6858), glycoproteins (*p*=0.6377), proteoglycans (*p*=0.8293), ECM-affiliated proteins (*p*=0.3368), secreted factors (*p*=0.1720), or intracellular proteins (*p*=0.6325). The EDC-TDM scaffolds had more ECM-regulators than the PLA (*p*=0.0119) or TDM (*p*=0.0380) scaffolds.

Within each treatment group, for seeded and unseeded scaffolds, collagens were the most abundant category by weight for all groups except the PLA unseeded (Figures 3(a) and 3(b) and Table 3). In all seeded scaffolds, collagens were the most abundant type of protein. The PLA-seeded scaffolds did not have significantly more collagens than glycoproteins (*p*=0.0514) or nonmatrisomal proteins (*p*=0.8730) (Table 3), while the both the TDM- and EDC-TDM-seeded scaffolds had significantly more collagens than any other protein type at Day 28 (Table 3).

The hASC-seeded scaffolds produced more glycoproteins (*p* < 0.001), ECM regulators (*p* < 0.0001), and ECM-affiliated (*p* < 0.0001) than were found in the unseeded PLA, TDM, and EDC-TDM scaffolds (Figure 3(c)). There was no difference between seeded and unseeded TDM (*p*=0.1697) or EDC-TDM (*p*=0.3105) scaffolds in proteoglycan content, but PLA-seeded scaffolds had greater proteoglycan content than the unseeded PLA (*p* < 0.001). Total collagens were also not different between seeded and unseeded for TDM (*p*=0.7764) and EDC-TDM (*p*=0.7897) scaffolds but were greater in seeded PLA scaffolds compared with unseeded PLA scaffolds (*p*=0.0018). Nonmatrisomal proteins in all seeded groups and the TDM- and EDC-TDM-unseeded scaffolds all differed from the PLA unseeded (*p* < 0.0001).

#### 3.5.3. Collagens

**Figure 4:**
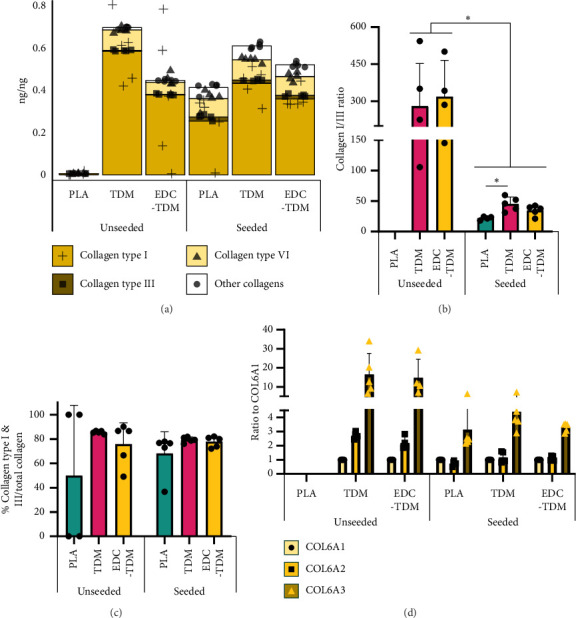
Collagen content in hASC-seeded meltblown scaffolds. Mean percentage of major collagens (a), collagen type I to collagen type III ratio (b), mean ± SD percent collagen types I and III of total collagens (c), and ratio of COL6A2 and COL6A3 to COL6A1 (d) for unseeded and hASC-seeded, untreated (PLA), tendon-derived matrix-coated (TDM), and TDM-crosslinked (EDC-TDM) poly(lactic acid) meltblown scaffolds after 28 days in culture. (*n* = 5; ^∗^*p* < 0.05, ^∗^^∗^*p* < 0.01, ^∗∗∗^*p* < 0.001, ^∗∗∗∗^*p* < 0.0001; ANOVA; Tukey's post hoc test). Significantly different collagens can be found in Table 3.

Collagen type I was the predominant collagen found in all treatment groups (Figure 4(a)), followed in abundance by collagen type VI, collagen type XII, and collagen type III. A total of 14 members of the collagen superfamily were found within the samples. At Day 28, the seeded, TDM-coated scaffolds had a higher collagen type I content than PLA scaffolds (*p*=0.0417), but EDC-TDM was not different to PLA (*p* > 0.05) (Table 2). In seeded scaffolds for PLA only, the collagen type I to collagen type III ratio was 21.94 ± 2.80, which was lower than the TDM-seeded scaffolds (45.22 ± 11.40) but not the EDC-TDM scaffolds (34.68 ± 8.43) (Figure 4(b)). All seeded scaffolds had a lower collagen type I to collagen type III ratio than the unseeded TDM (280.55 ± 172.25) and EDC-TDM scaffolds (318.76 ± 147.12) at Day 28 (*p* < 0.0001). Types I and III collagens made up 58%–73% of all collagens across the treatment groups (Figure 4(c)), but there was no effect of treatment (*p*=0.1166). Within collagen type VI, the COL6A1:COL6A2:COL6A3 ratio skewed heavily toward COL6A3, with 3-4.5-fold the COL6A3 compared with COL6A1 in seeded scaffolds (Figure 4(d)) and 15–17x higher than COL6A1 in the unseeded TDM and EDC-TDM scaffolds.

#### 3.5.4. Glycoproteins

**Figure 5:**
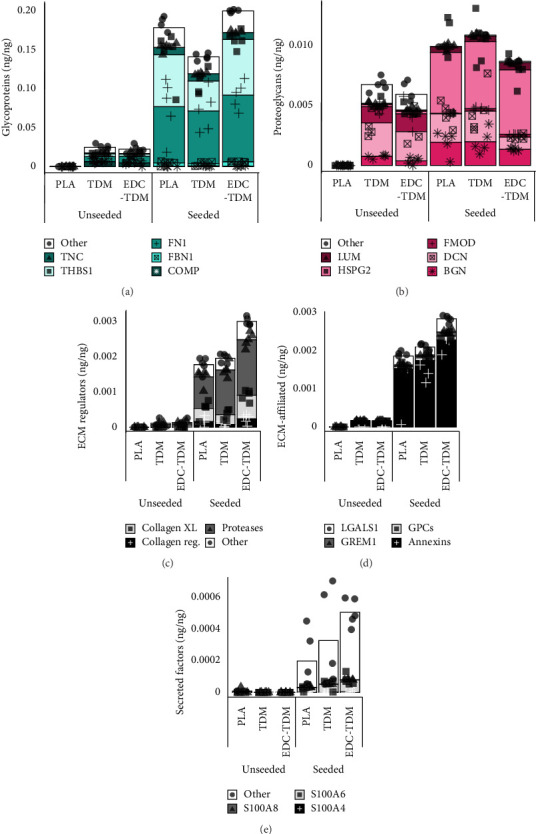
Glycoprotein, proteoglycan, and matrisome-affiliated protein content in hASC-seeded meltblown scaffolds. Mean percentage of major tendon-related glycoproteins (a), proteoglycans (b), major extracellular matrix (ECM) regulators (c), ECM-affiliated proteins (d), and secreted factors (e) for unseeded and hASC-seeded, untreated (PLA), tendon-derived matrix-coated (TDM), and TDM-crosslinked (EDC-TDM) poly (lactic acid) meltblown scaffolds after 28 days in culture. Significantly different matrisome-affiliated proteins can be found in Table 3.

All treatment groups produced tendon-associated glycoproteins, COMP, fibrillin 1 (FBN1), fibronectin (FN1), thrombospondin-1 (THSB1), and tenascin-C (TNC) (Figure 5(a)). In the seeded scaffolds, FN1 and THSB1 dominated the glycoprotein expression. The TDM-coated scaffolds had significantly more CILP2, COMP, and thrombospondin 4 (THBS4) expressions than the PLA scaffolds (Table 2), and the crosslinking of the TDM with EDC-TDM increased CILP2, NID2, SPARC, and EDIL3 expressions compared with PLA. Crosslinking the TDM did not result in any differences in glycoprotein expression between TDM and EDC-TDM scaffolds.

#### 3.5.5. Proteoglycans

hASC-seeded scaffolds produced the tendon-associated proteoglycans biglycan (BGN), decorin (DCN), fibromodulin (FMOD), lumican (LUM), and the pericellular matrix protein perlecan (HSPG2) (Figure 5(b)). TDM treatment or EDC crosslinking the TDM did not cause a difference in biglycan (*p*=0.4094) or decorin (*p*=0.1345) synthesis compared with PLA only scaffolds at Day 28. The TDM-coated scaffolds had increased fibromodulin compared with the PLA scaffolds (Table 2), and EDC-TDM scaffolds exhibited an increase in fibromodulin and PRELP compared with PLA. Crosslinking the TDM with EDC did not lead to any differences in proteoglycan expression between the TDM and EDC-TDM scaffolds.

#### 3.5.6. Matrisome-Affiliated Proteins

hASC-seeded PLA, TDM, and EDC-TDM scaffolds expressed collagen regulators, collagen crosslinkers—including LOXL2—and a variety of proteases (Figure 5(c)). TDM and EDC-TDM groups both demonstrated increased MMP2 compared with PLA scaffolds (Table 2). The EDC-TDM scaffolds had increased LOXL2 expression compared with both PLA and TDM scaffolds. All groups also highly expressed annexins and glypicans, as well as GREM1 and LGALS1 at 28 days in culture (Figure 5(d)). EDC-TDM scaffolds had increased expression of annexin 6 compared with PLA scaffolds and GREM1 compared with PLA and TDM scaffolds (Table 2). The hASCs in all groups secreted S100 calcium binding proteins A4 (S100A4), A6 (S100A6), and A8 (S100A8), with S100A6 being predominant (Figure 5(e)). EDC-TDM scaffolds had increased S100A6 compared with PLA scaffolds (Table 1).

#### 3.5.7. Human vs. Porcine Specific Proteins

**Figure 6:**
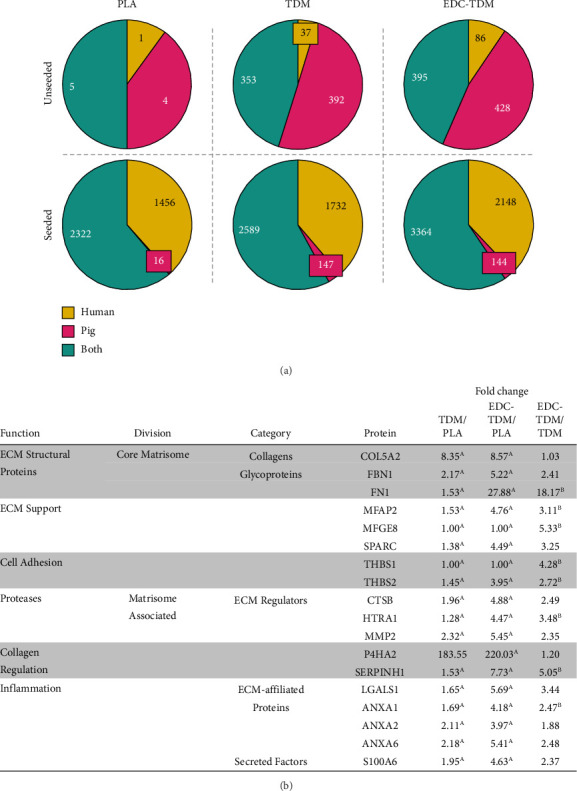
Peptide mapping to human and pig proteome. Number of peptides (a) that mapped to the human (*Homo sapiens*), pig (*Sus scrofa*), or equally to both. Differentially expressed proteins (b) that mapped only to human (Homo sapiens) reference proteome. Letters = significance: ^A^> PLA, ^B^> TDM (*p*  <  0.05, ANOVA; Tukey's post hoc test, *n* = 5).

In order to assess the contribution of the porcine TDM coated onto the scaffolds at the beginning of the experiment compared with new human tendon-like extracellular matrix synthesized specifically by hASCs in response to the scaffolds, we compared the overall peptide dataset for match only to human (ID: UP000005640) or only to porcine (ID: UP000008227) reference proteomes in BLAST [54] or to both. Peptides were assigned to human, pig, or both based on percent identity of the top hit: if a peptide had a greater percent identity to a protein from the human reference proteome, it was assigned “human”; if a peptide had a greater percent identity to a protein from the porcine reference proteome, it was assigned “pig”; and if a peptide had equal percent identity to a human and porcine protein, it was assigned as “both”. Of the 8887 total peptides identified, 3369 peptides (37.9%) mapped to the human reference proteome only, 687 peptides (7.7%) mapped to the pig reference proteome only, and 4831 peptides (54.4%) mapped to both (Supporting Table 11), with equal percent identity assigned to human and pig proteins (Supporting Table 12). As expected, the TDM and EDC-TDM unseeded scaffolds had mostly pig-only identifications, with similar number of peptides that mapped to both human and pig (Figure 6(a)). We expected the peptides that mapped to both human and pig in the unseeded TDM samples came from pig proteins because they contained porcine TDM only and no human-derived material. For the seeded scaffolds, PLA scaffolds had 3794 unique peptides, TDM scaffolds had 4468 unique peptides, and EDC-TDM scaffolds had 5656 unique peptide identifications. These peptide identifications predominantly mapped toward both human and pig or human only, with few peptides mapping only to pig (Figure 6(a)). At Day 28, the TDM seeded scaffolds contained 37.5% of the pig only identifications compared with unseeded TDM, while EDC-TDM scaffolds contained 33.6% of the pig-only identifications. Of the peptides that matched only to *Homo sapiens*, i.e., definitively produced by the hASCs, 8 core matrisome proteins and 10 matrisome-affiliated proteins were differentially expressed by fold-change intensity analysis (Supporting Table 13 and Figure 6(b)). EDC-TDM-crosslinked scaffolds had an increase in MMP2 compared with PLA scaffolds. Crosslinking the TDM with EDC led to an increase in FN1, MFAP2, SPARC, THBS1, CTSB, P4HA2, LGALS1, and S100A6 compared with the uncrosslinked TDM scaffolds.

## 4. Discussion

Incorporating TDM, with or without crosslinking it onto PLA meltblown scaffolds, had no effect on the rate of matrix synthesis of hASCs grown on meltblown scaffolds or on collagen alignment, but TDM and EDC-TDM had proteomic evidence of an increase in maturity of the engineered tendon extracellular matrix. The TDM also showed benefit with improved yield stretch and stress of the scaffolds at Day 28 whereas EDC-TDM showed only maintenance of Day 0 mechanical properties at Day 28 instead of the rapid degradation observed in unseeded scaffolds.

All treatment groups developed tendon-like matrisomes after 28 days in culture: high levels of structural collagen, with high amounts of glycoproteins—fibronectin particularly, and glycosaminoglycans. Interestingly, carbodiimide crosslinking led to an increase in collagen regulators, proteases, and crosslinkers that suggest increased matrix remodeling and/or maturation stimulated specifically by the crosslinking process in the de novo tendon-like proteome. However, overall the extracellular matrix appeared to be ‘immature' in the engineered tendon compared to that which would be expected of a mature adult tendon phenotype based on proportions of collagen types I and III, but confirmation of this awaits morphometric study of the newly formed collagen fibrils. Tendon is comprised of 60%–85% collagen by dry weight [55]. The collagen type I content of Day 28 hASC-seeded meltblown scaffolds was between the collagen type I content in patellar tendon (∼82%) and anterior cruciate ligament (∼53%) [52]. Collagen type III comprised 1.1%–1.5% of the quantified proteome in seeded scaffolds, which is lower than available estimates for native tendon but still within expected values [52, 55–57]. The collagen type I to III ratio in all Day 28 seeded meltblown scaffolds ranged from ∼22 to 45, which is similar to the collagen type I to III ratio in pig Achilles' tendon (∼30) [58] but greater than measured in human patellar tendon (13.28 ± 3.86) [52] or human supraspinatus tendon (∼2.5–4) [59]. The collagen type I to III ratio varies by the tendon type, and the TDM increased the collagen type I to III ratio compared with PLA scaffolds alone. The TDM used was prepared from a mix of pig hindlimb tendons—primarily flexor tendons. The increased collagen type I/III ratio in seeded TDM scaffolds could be a result of the tendons used to prepare TDM rather than a more mature matrix, and these possibilities could not be excluded during analysis of the porcine compared to human peptides.

Collagen types I and III colocalize in fascicles during early tendon development but as it develops, collagen type III regulates collagen type I fibrillogenesis and moves to the interfasicular matrix, leading to an increasing collagen type I/III ratio as the tendon matures [60–63]. We have previously observed a similar localization of collagen type III in a collagen gel based-tendon and ligament matrix powder scaffold, whereas collagen type I was located throughout the construct [41]. However, in our prior electrospun work, similar differential localization of collagen type I and type III was not observed [10]. While collagen type III serves as a provisional scaffold in wound healing and paves the way for collagen type I deposition [63], gene ontology analysis did not provide evidence for a fibrosis- or reparative-type proteome, instead there was evidence for upregulation of tendon developmental pathways. Potentially, the scaffolds need longer than 28 days in culture or mechanical stimulation [64] to form a more mature engineered tendon proteome. After 28 days in culture, the unseeded TDM and EDC-TDM scaffolds had 0.3% and 0.2% collagen type III by weight, leading to high collagen type I to III ratios. We did not quantify the proteome of the TDM before application to scaffolds in this study but suspect that collagen type III was not retained in the unseeded scaffolds during coating and seeding.

All seeded scaffolds had high amounts of the glycoproteins THSB1 and FN1, which, while found in tendon [55], are not as prominent as identified in the engineered tendon here. THSB1 overexpression in ASCs inhibits angiogenesis and osteogenesis in tissue engineered cartilage [65]. Recombinant THSB1 exhibits a prochondrogenic effect on bone-marrow derived MSCs, which do have a higher differentiation potential toward chondrocytes than ASCs [66]. The high levels of THSB1 identified here could represent either a phenotypic drive towards the relatively hypovascular phenotype of tendon, direction of engineered tendon development away from osteogenic lineages, or even possibly away from an enthesis-type phenotype toward more tendon proper. During development, fibronectin is initially expressed at high levels—particularly in the myotendinous junction, where the fibronectin-rich matrix attaches tendon to muscle before being replaced by a collagen-rich matrix [16, 67, 68]. Fibronectin expression is higher in ligament than in tendon [52, 69] and increases in fibrotic tendon after injury [70], although that increase is often brief [71]. High concentrations of fibronectin in fetal bovine serum could also lead to an increase in fibronectin receptors in the cells during a long-term culture [71], like the 10% fetal bovine serum we used to culture our cells in this study, although this phenomenon is not fully understood. Fibronectin is often the first ECM protein assembled into fibers [72, 73] and can act as a provisional scaffold for assembling the collagen fiber matrix [74–77]. Fibronectin promotes cell migration, alignment and spreading [78, 79], which could help the cells infiltrate the scaffold (as seen in Figure 1) before beginning to replace the fibronectin-rich matrix with a collagen-rich tendon matrix. Meltblown scaffolds exhibit similar cell infiltration and matrix production to single layer electrospun scaffolds produced with the use of sacrificial fibers but over a shorter timespan (28 days vs. 12 weeks) [80]. A longer culture period could determine whether the hASCs in our scaffolds replace the high levels of fibronectin with a more collagen-rich, mature tendon-like matrix.

While many tendon-related proteoglycans and glycoproteins were identified in the neo-matrix, several common tendon-related proteins, notably elastin, versican, aggrecan, and tenomodulin were missing. Elastin makes up 2% of tendon by dry weight [81], where the elastic fibers are highly localized near tenocytes, around and in series with them [82–84]. The role of elastin in tendon is not fully understood but seems to support collagen crimp, tendon prestress and the toe-region response, as well as transverse and shear loading [85, 86]. Elastin gene expression and protein synthesis occurs primarily during late embryonic development, but not during adult life [87], and transforming growth factor beta 1 (TGF-β1) upregulates tropoelastin gene expression [88]. Our use of adult stem cells (ages 36–59) and no exogenous growth factors like TGF-β1 in the culture media could both determine the lack of elastin expressed. Versican and aggrecan both have roles in collagen fibrillogenesis, with versican promoting larger collagen fibril formation and aggrecan playing the opposite role [89]. Low levels of aggrecan and versican are seen in the Achilles' tendon and the anterior cruciate ligament but not in the patellar tendon [52, 90]. The expression of both aggrecan and versican are regulated by mechanics: aggrecan tends to appear in compressive regions of tendon [91] while versican appears in tendons bearing primarily tensile loads [92], and both increase with age [92], thus the lack of these proteoglycans in the engineered tendon produced here is not surprising, given the lack of loading or loading history present in these culture conditions. Tenomodulin is a transmembrane glycoprotein necessary for tendon development and maturation [93] and loss of tenomodulin exacerbates scar tissue formation [94]. Tenomodulin gene expression increases at Day 7 in hASCs cultured on aligned electrospun scaffolds [19], but the meltblown scaffolds used in this study are less aligned than the electrospun scaffolds [31] and fiber alignment affected tenomodulin gene expression substantially [19]. Currently, the extent of tenomodulin gene expression in hASCs cultured on meltblown scaffolds is unknown [31]. In addition, MSCs express much less tenomodulin than tendon-derived stem cells under similar conditions [95], and tensile stretch further promotes tenomodulin expression [96]. Together, since our scaffolds were cultured in static conditions for only 28 days, the combination of cell source, cell culture media, lack of loading, and the duration of culture could influence the lack of observed elastin, versican, aggrecan, and tendomodulin identified here.

In the Top3 quantification, comparing seeded PLA, TDM, and EDC-TDM scaffolds after 28 days in culture, we found 14 differentially expressed proteins between groups: 4 collagen, 7 glycoproteins, 3 proteoglycans, and 5 matrisome-affiliated proteins (Table 2)—when considering both pig and human proteins. Incorporating the TDM resulted in more collagen type I—the predominant collagen found in tendon [52]. However, that increase was lost in seeded EDC-TDM scaffolds compared with seeded PLA scaffolds when measured by Top3 quantification (Figure 4), even though the EDC crosslinking increased the total collagen as measured by hydroxyproline assay on meltblown (Figures 1(f), 1(g), and 1(h)) and previously on electrospun scaffolds [36]. The EDC-TDM-crosslinked scaffolds had more glycoproteins and ECM regulators than the TDM scaffolds, which could account for the lower percentage of collagen type I. hASCs produce significantly more collagen (5–10X) on meltblown scaffolds compared with electrospun scaffolds [10, 19, 31, 36], which could dampen the beneficial effect of TDM on meltblown scaffolds. The TDM scaffolds demonstrated an increase in CILP2, COMP, THBS1, and FMOD, which are associated with tendon extracellular matrix [97, 98]—often with collagen fibrillogenesis [99] and structural support that is beneficial to tendon. On the other hand, EDC-TDM scaffolds had increased COMP, THBS1, and PRELP expression, which are more highly expressed in the anterior cruciate ligament than in patellar tendon [52]. This suggests that incorporating the TDM is promoting development of a collagen-rich matrix. Recent work suggests that COMP and CILP2 are markers of a highly differentiated fibroblast stage named a matrifibrocyte in postmyocardial infarction scarring, cells that are highly differentiated towards matrix production in highly collagenous environments, suggesting that increased COMP and CILP2 could represent greater neomatrix maturity [100]. Similarly, elevations in periostin in TDM-treated scaffolds could represent a marker of extracellular reorganization and fibroblast activation [101]. Thus, there may be a critical balance between the more quiescent hASC- differentiated tendon fibroblast lineage, and a more active tendon fibroblast lineage, but these studies await single cell sequencing and spatial proteomics. Both the TDM and EDC-TDM samples exhibited an increase in MMP2, which is associated with tendon overuse [102] but also with tendon remodeling. The fibronectin rich matrix in our scaffolds could promote MMP2 expression [103], which cleaves the fibronectin into small fragments and promotes cell adhesion and migration [104]. Overall, incorporating TDM into meltblown scaffolds promoted a more mature tendon-like matrix than PLA alone.

EDC-TDM-crosslinked scaffolds exhibited an increase in CILP2, NID2, SPARC, EDIL3, GREM1, LOXL2, ANXA6, and S100A6 (Table 1). SPARC is necessary in tendon development [105] and associated with more mature tendon [106]. NID2 is a basement membrane protein that promotes collagen binding [107]. EDIL3 regulates integrin binding [108]. NID2 and EDIL3 are more frequently associated with cartilage than tendon, but both protect chondrocytes against osteoarthritis [109–112]. Interestingly, overexpression of both proteins are related to cancer tumor progression and invasion [113, 114] but could have a similar role here as the cells infiltrate the scaffolds. Higher levels of GREM1 and S100A6, however, correlate with osteoarthritis [115, 116], opposing the expression of NID2 and EDIL3. TDM incorporation led to an increase in expression of proteins that regulate the pro- and anti-inflammatory balance. S100A6, Annexins A1, A2, and A6 are calcium-binding proteins associated with inflammation [117–120]. While Annexin A1 has an anti-inflammatory role in tendon [121], Annexin A2 is associated with increased proadipogenic inflammation in a rat rotator cuff injury model [118]. In addition, LGALS1—also increased when TDM is incorporated—is proinflammatory in osteoarthritis via activation of the NF-κB pathway [122]. S100A4 is a driver of the fibrotic response in tendon [123], and S100A6 has a similar role in liver fibrosis [124], although the role of S100A6 in tendon, if any, is unknown. S100A6 is a fibroblast marker [125] and is expressed by ASCs in culture, where it is frequently upregulated during proliferation and growth [126, 127]; therefore, it is unlikely to be directly proinflammatory in tendon. GREM1 serves as a BMP antagonist [128], which could prevent differentiation of the hASCs into bone. Taken together, EDC-TDM-crosslinked scaffolds promoted a more mature matrix than the PLA or TDM scaffolds, with more matrix remodeling proteins expressed. Together, the data suggest that besides development of a tendon matrisome, engineered tendon on meltblown scaffolds develops a molecular balance between pro- and antifibrotic and pro- and anti-inflammatory markers that could be critical to success of downstream translational applications.

In the EDC-TDM scaffolds, LOXL2, a collagen crosslinker [129], was upregulated compared with both the PLA and TDM scaffolds. LOXL2 is a paralog of LOX, which is an important regulator of tendon elastic modulus during development, with increased LOX-mediated hydroxylysyl and lysyl pyridinoline crosslinks correlating directly with elastic modulus [130, 131]. LOXL2 is generally better studied in cartilage than tendon and plays an important role in chondrogenesis [132]. While LOXL2 crosslinking increases pyridinoline crosslinks, this did not result in improved compressive modulus of engineered bone graft constructs after 28 days in culture [133]. Similarly, in our study, the increased LOXL2 expression was not associated with improvement in tensile properties of EDC-TDM compared with TDM at 28 days.

When we considered the de novo expression of definitively human-only proteins, incorporating TDM or crosslinking the TDM with EDC saw increased expression of 19 proteins total (Table 2). Collagen type V, while a low abundance collagen in tendon, has an important role in collagen type I nucleation and fibril diameter [134–136]. Thrombospondins 1 and 2 were both upregulated, and both play roles in collagen fibrillogenesis [137, 138], while MFGE8 regulates collagen endocytosis and helps remove excess collagen in the extracellular space [139, 140]. Fibronectin and fibrillin 1 are predominantly known for functioning as ECM structure and support [141–143]. Fibrillin 1 forms microfibrils in tendon that colocalize with elastin fibers within the collagen matrix, supporting tendon mechanics [141]. Fibrillin 1 expression was higher in tendon than ligament with the opposite occurring for fibronectin [52], but both were increased in hASCs when TDM was incorporated into the scaffold, perhaps acting as a template for future elastin deposition. MFAP2 is often associated with elastin microfibrils but also supports fibrillin 1 microfibrils [144], which is likely its role in our engineered tendon. While no elastin was identified in cultured scaffold, de novo expression of elastin-associated proteins was associated with incorporation of TDM, and these elastin-associated proteins could ‘prime' the engineered tendon for expression and organization of elastin, when required. Thus, TDM enhanced the expression of proteins associated with normal collagen and elastin fibrillogenesis.

Three proteases were upregulated by incorporating TDM. CTSB is a thiol protease that cleaves glycoproteins in the basement membrane [145] and enables collagen binding [146]. HTRA1 is a serine protease that targets fibronectin, decorin, and fibromodulin and can lead to MMP3 upregulation [147, 148]. Serpin Family H Member 1 (SERPINH1) acts as a chaperone for collagen molecules during collagen synthesis [149] and P4HA2 is a key enzyme in catalyzing the 4-hydroxyproline that allows the proper folding of newly synthesized collagen into fibrils [150]. Thus, the incorporation of TDM stimulated expression of matrix remodeling proteins.

Crosslinking the TDM on meltblown scaffolds lead to an increase in total collagen (by hydroxyproline assay) when cultured in optimal tendon media enriched in L-ascorbate (2.3, Figure 1(h)), as found in our prior work with hASCs cultured on aligned electrospun scaffolds [36]. However, using media optimized for noncanonical amino acid labeling did not result in higher total collagen content as measured by hydroxyproline assay (Supporting Figures 2C and 2F). Similarly, Top3 quantification of the proteome (Figure 3(c)) of scaffolds cultured in optimal tendon media did not confirm the hydroxyproline assay identified increase in the collagen content.

The EDC-TDM scaffolds saw no benefit to mechanical properties over culture, despite EDC crosslinking previously being associated with increased collagen stiffness and strength, albeit at molar ratios of 5:2:1 for EDC:NHS:COO^−^ [151]. EDC-crosslinking TDM coated on electrospun scaffolds trended toward increasing scaffold modulus and stiffness while UV crosslinking the TDM increased modulus and stiffness [36]. In both cases, mechanical properties were measured without performing cell culture [36, 151]. However, even the Day 0 unseeded EDC-TDM scaffolds had no increase in tangent modulus compared with PLA or TDM (Supporting Table 2). The meltblown scaffolds used here have ∼6–8 greater modulus and higher stiffness [31] than the electrospun scaffolds [36] and that higher initial modulus and stiffness could limit the benefit of EDC crosslinking the TDM on these meltblown scaffolds. A recent study suggested that electrospun fiber microarchitecture could be partially ‘masked' by a similar method of TDM application as that used here; we are currently optimizing alternate methods of TDM application and cross-linking to prevent these issues in the future [36]. The fiber diameter of the meltblown fabrics used in this study are almost a magnitude of order larger than the fiber diameters of the electrospun scaffolds used in our previous studies investigating TDM. Our findings suggest that the larger fiber diameter could stimulate some degree of tenogenesis without the need for exogenous biomimetic factors. A review of the tendon literature reveals that tendon research commonly investigates fiber diameters below 4 microns [152, 153], but our findings suggest that more knowledge is needed regarding how cells respond to a larger range of fiber diameters, which we plan in future studies.

## 5. Conclusions

Incorporating TDM by coating the scaffolds had benefits for scaffold mechanical function and both TDM and EDC-TDM promoted matrix expression of proteins to build a more mature, collagen-rich matrix than the PLA meltblown scaffold alone. Combined with our previous experience, these data confirm that TDM stimulates tenogenesis in a manner dependent on underlying scaffold structure and architecture as well as on biomimetic cues. These data suggest that further refinement of TDM application and further refinement of fiber parameters for tenogenesis would be beneficial in moving toward translation.

## Figures and Tables

**Table 1:** Differentially expressed matrisome proteins by intensity fold change in seeded Day 28 scaffolds.

Division	Category	Protein	Fold change		
			TDM/PLA	EDC-TDM/PLA	EDC-TDM/TDM
Core matrisome	Collagens	COL1A2	4.08	5.88^A^	1.44
		COL5A2	6.82^A^	5.77^A^	−1.18
	ECM glycoproteins	EDIL3	2.61	12.02^A^	4.61^B^
		MFAP2	1.86	5.33^A^	2.87
		MFGE8	1.40	4.49^A^	3.20
		SPARC			3.29
		THBS1	1.14	3.04^A^	2.67
		THBS2	1.88	4.38^A^	2.33

Matrisome associated	ECM regulators	CTSB	1.28	4.51^A^	3.51^B^
		HTRA1	2.24	5.75^A^	2.57
		LEPRE1	3.96	10.90^A^	2.75
		LOXL2	−1.02	5.11^A^	5.23^B^
		MMP2	183.55	220.03^A^	1.20
		P4HA2	1.78	6.80^A^	3.82
		PLOD2	2.09	5.61^A^	2.69
		TIMP3	4.62	9.48^A^	2.05
	ECM-affiliated proteins	ANXA1	1.79	4.57^A^	2.56^B^
		ANXA2	2.28	4.56^A^	2.00
		ANXA4	6.75	21.11^A^	3.13
		ANXA6	2.18	5.41^A^	2.48
		GREM1	1.63	9.83^A^	6.02^B^
		LGALS1	1.95	4.63^A^	2.37
	Secreted factors	ANGPTL7			5.25
		S100A6	2.16	8.51^A^	3.94^B^

*Note:* Letters = significance, ANOVA; Tukey's post hoc test, *n* = 5, *p* < 0.05.

^A^> PLA.

^B^> TDM

**Table 2:** Differentially expressed matrisome proteins by Top3 quantification.

Division	Category	Protein	Fold change		
			TDM/PLA	EDC-TDM/PLA	EDC-TDM/TDM
Core matrisome	Collagens	COL1A1	1.57^A^	1.44	−1.09
		COL1A2	1.35	−1.09	−1.47^B^
		COL5A2	11.10^A^	3.86	−2.87
		COL6A1	−1.67^A^	−1.52^A^	1.10
	Glycoproteins	CILP2	1.00^A^	1.00^A^	−1.03
		COMP	5.29	5.17	−1.02
		EDIL3	1.75	3.68^A^	2.11
		NID2	1.00	1.00^A^	3.72
		POSTN	−3.06^A^	−2.03^A^	1.50
		SPARC	1.00	1.00^A^	2.69
		THBS1	−2.29^A^	−1.25	1.82^B^
	Proteoglycans	BGN	1.00^A^	1.00	−2.98
		FMOD	4.14^A^	4.75^A^	1.15
		PRELP	13.37	18.40^A^	1.38

Matrisome-associated	ECM regulators	LOXL2	−1.58	2.45^A^	3.88^B^
		MMP2	74.01^A^	62.80^A^	−1.18
		TIMP3	1.46	2.84^A^	1.94
	ECM-affiliated proteins	ANXA4	6.90	15.18^A^	2.20

*Note:* Letters = significance, ANOVA; Tukey's post hoc test, *n* = 5, *p*  < 0.05.

^A^> PLA.

^B^> TDM

**Table 3:** Difference in Top3 Quantification by the protein category.

	ANOVA *p* value	Core matrisome			Matrisome affiliated			NM
		COL	GP	PG	ER	EA	SF	
Unseeded								
PLA	< 0.0001	B	B	B	B	B	B	A
TDM	< 0.0001	A	C	C	C	C	C	B
EDC-TDM	< 0.0001	A	BC	C	C	C	C	AB
Seeded								
PLA	< 0.0001	A	AB	B	B	B	B	A
TDM	< 0.0001	A	C	D	D	D	D	B
EDC-TDM	< 0.0001	A	C	D	D	D	D	B

*Note:* GP = ECM glycoproteins; Letters = significance: A (highest percentage) ⟶ *Z* (lowest percentage) (ANOVA; Tukey's post hoc test, *n* = 5), *p* < 0.05.

Abbreviations: COL, collagens; EA, ECM affiliated; ER, ECM regulators; NM, nonmatrisomal; PG, proteoglycans; SF, secreted factors.

## Data Availability

Processed proteomic datasets required to reproduce these findings are included in supplemental data. Raw proteomic datasets and other raw data are available on request from the corresponding author.

## Nomenclature

ADH: Alcohol dehydrogenase
AHA: L-Azidohomoalanine
ANGPTL7: Angiopoietin-like 7
ANOVA: Analysis of variance
ANXA1: Annexin A1
ANXA2: Annexin A2
ANXA4: Annexin A4
ANXA6: Annexin A6
BGN: Biglycan
CILP2: Cartilage intermediate layer protein 2
COL1A1: Collagen type I alpha 1
COL1A2: Collagen type I alpha 2
COL5A2: Collagen type V alpha 2
COL6A1: Collagen type VI alpha 1
COL6A2: Collagen type VI alpha 2
COL6A3: Collagen type VI alpha 3
COMP: Cartilage oligomeric matrix protein
CTSB: Cathepsin B
DCN: Decorin
EDC-TDM: Carbodiimide-crosslinked TDM coated
EDC: 1-ethyl-3-(3-dimethylaminopropyl) carbodiimide hydrochloride
EDIL3: EGF-like repeats and discoidin I-like domains 3
FBN1: Fibrillin 1
FMOD: Fibromodulin
FN1: Fibronectin
GREM1: Gremlin 1
hASCs: Human adipose-derived stem cells
HSPG2: Perlecan
HTRA1: HtrA serine peptidase 1
LEPRE1: Leucine proline-enriched proteoglycan
LGALS1: Lectin, galactoside-binding, soluble, 1
LOXL2: Lysyl oxidase-like 2
LUM: Lumican
Met: Methionine
MFAP2: Microfibrillar-associated protein 2
MFGE8: Milk fat globule-EGF factor 8 protein
MMP2: Matrix metallopeptidase 2
MSCs: Mesenchymal stem cells
NHS: N-hydroxysuccinimide
NID2: Nidogen 2
P4HA2: Prolyl 4-hydroxylase, alpha polypeptide II
PLA: Poly(lactic acid)
PLOD2: Procollagen-lysine, 2-oxoglutarate 5-dioxygenase 2
POSTN: Periostin, osteoblast specific factor
PRELP: Proline/arginine-rich end leucine-rich repeat protein
S100A4: S100 calcium binding protein A4
S100A6: S100 calcium binding protein A6
S100A8: S100 calcium binding protein A8
SERPINH1: Serpin Family H Member 1
sGAG: Sulfated glycosaminoglycan
SPARC: Secreted protein, acidic, cysteine-rich
TDM: Tendon-derived matrix
TGF-β1: Transforming growth factor beta 1
THBS1: Thrombospondin-1
THBS2: Thrombospondin-2
THBS4: Thrombospondin-4
TIMP3: Tissue metallopeptidase inhibitor 3
TNC: Tenascin-C

## Supporting Information

Supporting Information 1 Supporting Figure 1: Representative stress-stretch curves for PLA, tendon-derived matrix (TDM) coated, and TDM-coated carbodiimide crosslinked (EDC-TDM) meltblown scaffolds for Day 0 unseeded scaffolds (A), Day 0 hASC-seeded scaffolds (B), and Day 28 hASC-seeded scaffolds (C).

Supporting Information 2 Supporting Figure 2: Mean ± SD total protein (A), dsDNA (B), and collagen (C) content of PLA-, TDM-, and EDC-TDM AHA-fed scaffolds days measured using biochemical assays after imaging. Mean ± SD total protein (D), dsDNA (E), and collagen (F) content of PLA-, TDM-, and EDC-TDM methionine-fed scaffolds days measured using biochemical assays after imaging (^∗^*p* < 0.05, ^∗∗^*p* < 0.01, ^∗∗∗^*p* < 0.001, ^∗∗∗∗^*p* < 0.0001; ANOVA; Tukey's post hoc test, *n* = 5).

Supporting Information 3 Supporting Table 1: Comparison of Mechanical Properties of Seeded vs. Unseeded Scaffolds at Day 0 (*n* = 16 (Unseeded)–15 (Seeded)) and Day 28 (*n* = 5 (Unseeded)–14 (Seeded)); Wilcoxon test, *α* = 0.05.

Supporting Information 4 Supporting Table 2: Mean ± SD tangent modulus at 4% strain for PLA, tendon-derived matrix (TDM) coated, and TDM-coated, carbodiimide crosslinked (EDC-TDM) meltblown unseeded scaffolds at day 0 and hASC-seeded scaffolds at days 0 and 28 (*n* = 2–5/treatment/time point; no significant difference, ANOVA, *p* > 0.05).

Supporting Information 5 Supporting Table 3: Mean ± SD transition strain for PLA, tendon-derived matrix (TDM) coated, and TDM-coated, carbodiimide crosslinked (EDC-TDM) meltblown unseeded scaffolds at Day 0 and hASC-seeded scaffolds at Days 0 and 28 (*n* = 2–5/treatment/time point; no significant difference, ANOVA, *p* > 0.05).

Supporting Information 6 Supporting Table 4: List of all peptide sequences identified by mass spectrometry with intensity values per sample.

Supporting Information 7 Supporting Table 5: List of all proteins identified by mass spectrometry mapped to human (*Homo sapiens*) or pig (*Sus scrofa*) with protein description and intensity values per sample.

Supporting Information 8 Supporting Table 6: Matrisome analysis of proteins identified with intensity values and Top3 quantification values per sample. Proteins are categorized by the Matrisome Project Database [53].

Supporting Information 9 Supporting Table 7: Fold-change analysis of protein intensity in hASC-seeded, untreated (PLA), tendon-derived matrix (TDM) coated, and TDM-crosslinked (EDC-TDM) PLA meltblown scaffolds after 28 days in culture (ANOVA; Tukey's post hoc test, *n* = 5).

Supporting Information 10 Supporting Table 8: Fold-change analysis of Top3 quantification in hASC-seeded, untreated (PLA), tendon-derived matrix (TDM) coated, and TDM-crosslinked (EDC-TDM) PLA meltblown scaffolds after 28 days in culture (ANOVA; Tukey's post hoc test, *n* = 5).

Supporting Information 11 Supporting Table 9: Fold-change analysis of protein intensity between unseeded and hASC-seeded, untreated (PLA), tendon-derived matrix (TDM) coated, and TDM-crosslinked (EDC-TDM) PLA meltblown scaffolds after 28 days in culture (ANOVA; Tukey's post hoc test, *n* = 5).

Supporting Information 12 Supporting Table 10: Fold-change analysis of Top3 quantification between unseeded and hASC-seeded, untreated (PLA), tendon-derived matrix (TDM) coated, and TDM-crosslinked (EDC-TDM) PLA meltblown scaffolds after 28 days in culture (ANOVA; Tukey's post hoc test, *n* = 5).

Supporting Information 13 Supporting Table 11: List of peptide sequences and species mapping to human (*Homo sapiens*) or pig (*Sus scrofa*).

Supporting Information 14 Supporting Table 12: Results of BLAST analysis comparing the percent identity to human (*Homo sapiens*) or pig (*Sus scrofa*) for each peptide that mapped to both species.

Supporting Information 15 Supporting Table 13: Fold-change analysis of human (*Homo sapiens*)-only protein intensity in hASC-seeded, untreated (PLA), tendon-derived matrix (TDM) coated, and TDM-crosslinked (EDC-TDM) PLA meltblown scaffolds after 28 days in culture (ANOVA; Tukey's post hoc test, *n* = 5).

Supporting Information 16 Supporting Table 14: List of Gene Ontology biological process terms for significant for non-matrisomal proteins in seeded scaffolds identified by intensity fold change analysis (Supporting Table 7).

Supporting Information 17 Supporting Table 15: List of Gene Ontology biological process terms for significant for non-matrisomal proteins in seeded scaffolds identified by Top3 quantification fold change analysis (Supporting Table 8).

Supporting Information 18 Supporting Table 16: List of Gene Ontology biological process terms for significant for non-matrisomal proteins in tendon-derived matrix (TDM) coated seeded scaffolds compared to unseeded TDM scaffolds identified by intensity fold change analysis (Supporting Table 7).

Supporting Information 19 Supporting Table 17: List of Gene Ontology biological process terms for significant for nonmatrisomal proteins in TDM-crosslinked (EDC-TDM) PLA-seeded scaffolds compared with unseeded EDC-TDM scaffolds identified by intensity fold change analysis (Supporting Table 7).

Supporting Information 20 Supporting Table 18: List of Gene Ontology biological process terms for significant non-matrisomal proteins in tendon-derived matrix (TDM) coated seeded scaffolds compared with unseeded TDM scaffolds identified by intensity fold change analysis (Supporting Table 8).

Supporting Information 21 Supporting Table 19: List of Gene Ontology biological process terms for significant for non-matrisomal proteins in TDM-crosslinked (EDC-TDM) PLA-seeded scaffolds compared to unseeded EDC-TDM scaffolds identified by Top3 fold change analysis (Supporting Table 8).
