# Supplementary material for: Application of Tendon-Derived Matrix and Carbodiimide Crosslinking Matures the Engineered Tendon-Like Proteome on Meltblown Scaffolds
Source: J Tissue Eng Regen Med. 2025 Feb 26;2025:2184723. doi: 10.1155/term/2184723 (PMC11985250; doi:10.1155/term/2184723)
Supplement: Supporting Information 1 — Supporting Figure 1: Representative stress-stretch curves for PLA, tendon-derived matrix (TDM) coated, and TDM-coated carbodiimide crosslinked (EDC-TDM) meltblown scaffolds for Day 0 unseeded scaffolds (A), Day 0 hASC-seeded scaffolds (B), and Day 28 hASC-seeded scaffolds (C). [file 2184723.f1.pptx]

## Slide 1
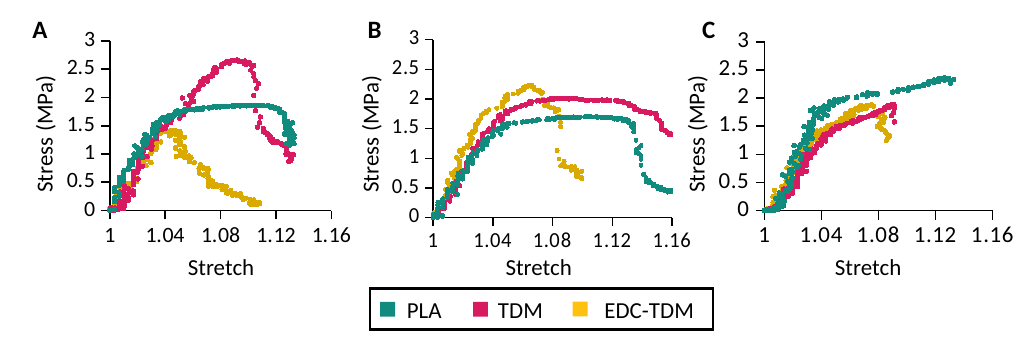

A
B
C
### Chart
| Category | | | |
|---|---|---|---|
### Chart
| Category | | | |
|---|---|---|---|
### Chart
| Category | | | |
|---|---|---|---|Stress (MPa)
Stress (MPa)
Stress (MPa)
Stretch
Stretch
Stretch
PLA
TDM
EDC-TDM
