# Supplementary figures and images for: Application of Tendon-Derived Matrix and Carbodiimide Crosslinking Matures the Engineered Tendon-Like Proteome on Meltblown Scaffolds
Source: J Tissue Eng Regen Med. 2025 Feb 26;2025:2184723. doi: 10.1155/term/2184723 (PMC11985250; doi:10.1155/term/2184723)

## Slide 1
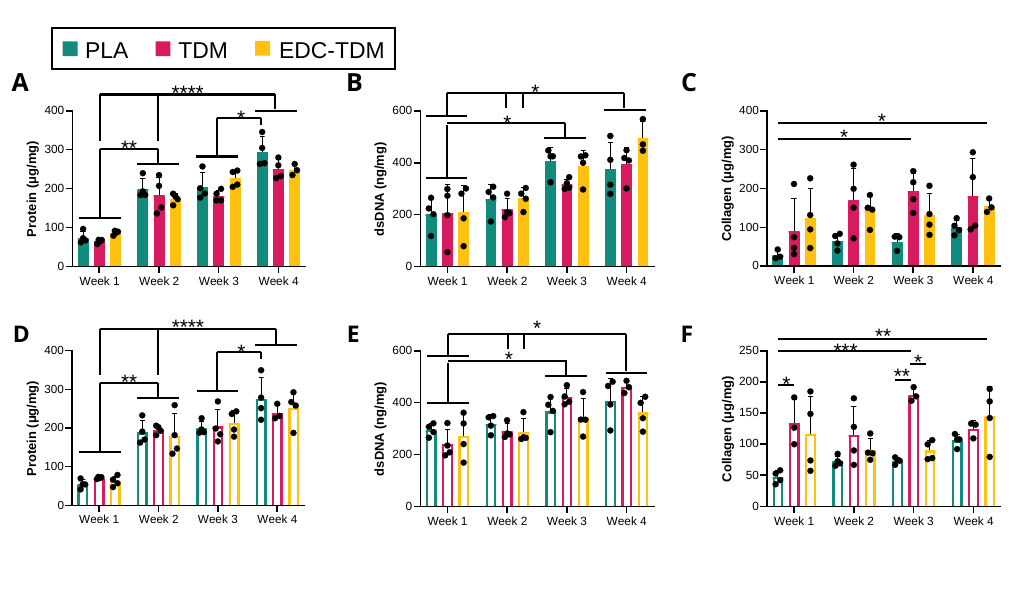

PLA
TDM
EDC-TDM
A
B
C
*
****
*
**
*
*
*
****
*
**
*
D
E
F
**
***
*
*
**
*

Supplement: Supporting Information 2 — Supporting Figure 2: Mean ± SD total protein (A), dsDNA (B), and collagen (C) content of PLA-, TDM-, and EDC-TDM AHA-fed scaffolds days measured using biochemical assays after imaging. Mean ± SD total protein (D), dsDNA (E), and collagen (F) content of PLA-, TDM-, and EDC-TDM methionine-fed scaffolds days measured using biochemical assays after imaging (∗p < 0.05, ∗∗p < 0.01, ∗∗∗p < 0.001, ∗∗∗∗p < 0.0001; ANOVA; Tukey's post hoc test, n = 5). [file 2184723.f2.pptx]
