# Supplementary material for: Application of Tendon-Derived Matrix and Carbodiimide Crosslinking Matures the Engineered Tendon-Like Proteome on Meltblown Scaffolds
Source: J Tissue Eng Regen Med. 2025 Feb 26;2025:2184723. doi: 10.1155/term/2184723 (PMC11985250; doi:10.1155/term/2184723)
Supplement: Supporting Information 3 — Supporting Table 1: Comparison of Mechanical Properties of Seeded vs. Unseeded Scaffolds at Day 0 (n = 16 (Unseeded)–15 (Seeded)) and Day 28 (n = 5 (Unseeded)–14 (Seeded)); Wilcoxon test, α = 0.05. [file 2184723.f3.docx]

**Additional Table 1**: Comparison of Mechanical Properties of Seeded vs Unseeded Scaffolds at Day 0 (n=16 (Unseeded) - 15 (Seeded)) and Day 28 (n=5 (Unseeded) - 14 (Seeded)); Wilcoxon test, α=0.05.

| **Time** | **Property** | Unseeded | Seeded | p-value |
| --- | --- | --- | --- | --- |
| Day 0 | Modulus (MPa) | **46.6 ± 20.0** | 32.4 ± 11.2 | **0.0378** |
|  | Yield Stretch | 1.04 ± 0.02 | 1.05 ± 0.02 | 0.6367 |
|  | Yield Stress (MPa) | **1.85 ± 0.38** | 1.28 ± 0.57 | **0.0062** |
|  | Stiffness (N/mm) | **21.3 ± 7.2** | 13.8 ± 4.6 | **0.0004** |
| Day 28 | Modulus (MPa) | **65.6 ± 19.9** | 40.1 ± 15.2 | **0.0074** |
|  | Yield Stretch | 1.03 ± 0.02 | **1.10 ± 0.06** | **0.0031** |
|  | Yield Stress (MPa) | 1.78 ± 0.70 | **3.13 ± 1.14** | **0.0169** |
|  | Stiffness (N/mm) | **22.6 ± 7.5** | 12.9 ± 5.5 | **0.0094** |
