# Supplementary material for: Application of Tendon-Derived Matrix and Carbodiimide Crosslinking Matures the Engineered Tendon-Like Proteome on Meltblown Scaffolds
Source: J Tissue Eng Regen Med. 2025 Feb 26;2025:2184723. doi: 10.1155/term/2184723 (PMC11985250; doi:10.1155/term/2184723)
Supplement: Supporting Information 4 — Supporting Table 2: Mean ± SD tangent modulus at 4% strain for PLA, tendon-derived matrix (TDM) coated, and TDM-coated, carbodiimide crosslinked (EDC-TDM) meltblown unseeded scaffolds at day 0 and hASC-seeded scaffolds at days 0 and 28 (n = 2–5/treatment/time point; no significant difference, ANOVA, p > 0.05). [file 2184723.f4.docx]

**Additional Table 2**: Mean±SD tangent modulus at 4% Strain for poly(lactic acid) (PLA), tendon-derived matrix coated (TDM), and TDM-coated, carbodiimide crosslinked (EDC-TDM) meltblown unseeded scaffolds at day 0 and hASC-seeded scaffolds at days 0 and 28 (n=2-5/treatment/time point; no significant difference, ANOVA, p>0.05).

| **Tangent Modulus (MPa)** | | PLA | TDM | EDC-TDM |
| --- | --- | --- | --- | --- |
| Unseeded | Day 0 | 29.94 ± 2.48 | 37.11 ± 3.08 | 32.83 ± 21.96 |
| Seeded | Day 0 | 23.48 ± 18.74 | 29.92 ± 0.81 | 31.05 ± 18.07 |
|  | Day 28 | 32.49 ± 18.20 | 27.43 ± 7.49 | 24.89 ± 9.87 |
