# Supplementary material for: Application of Tendon-Derived Matrix and Carbodiimide Crosslinking Matures the Engineered Tendon-Like Proteome on Meltblown Scaffolds
Source: J Tissue Eng Regen Med. 2025 Feb 26;2025:2184723. doi: 10.1155/term/2184723 (PMC11985250; doi:10.1155/term/2184723)
Supplement: Supporting Information 5 — Supporting Table 3: Mean ± SD transition strain for PLA, tendon-derived matrix (TDM) coated, and TDM-coated, carbodiimide crosslinked (EDC-TDM) meltblown unseeded scaffolds at Day 0 and hASC-seeded scaffolds at Days 0 and 28 (n = 2–5/treatment/time point; no significant difference, ANOVA, p > 0.05). [file 2184723.f5.docx]

**Additional Table 3**: Mean±SD transition strain for poly(lactic acid) (PLA), tendon-derived matrix coated (TDM), and TDM-coated, carbodiimide crosslinked (EDC-TDM) meltblown unseeded scaffolds at day 0 and hASC-seeded scaffolds at days 0 and 28 (n=2-5/treatment/time point; no significant difference, ANOVA, p>0.05).

| **Transition Stretch** | | PLA | TDM | EDC-TDM |
| --- | --- | --- | --- | --- |
| Unseeded | Day 0 | 0.999 ± 0.005 | 1.001 ± 0.004 | 1.009 ± 0.017 |
| Seeded | Day 0 | 1.005 ± 0.007 | 1.010 ± 0.008 | 1.006 ± 0.009 |
|  | Day 28 | 1.003 ± 0.004 | 1.010 ± 0.007 | 1.009 ± 0.009 |
